# Gambling behavior among Macau college and university students

**DOI:** 10.1186/s40405-017-0022-7

**Published:** 2017-04-04

**Authors:** Sut Mei Kam, Irene Lai Kuen Wong, Ernest Moon Tong So, David Kin Cheong Un, Chris Hon Wa Chan

**Affiliations:** 1 Yat On Responsible Gambling Counseling Centre, Macau, China; 2 Faculty of Health and Social Sciences, Hong Kong Polytechnic University, Hong Kong, China; 3 Department of Sociology, University of Hong Kong, Hong Kong, China

**Keywords:** Problem gambling, Affect states, Sensation seeking, Macau students

## Abstract

This survey investigated gambling behavior among Chinese students studying in Macau colleges and universities. It also aimed to examine the relationship between problem gambling, affect states and sensation seeking propensity. A convenience sample of 999 students (370 men, 629 women) filled a self-administered questionnaire consisted of the Problem Gambling Severity Index (PGSI) (Ferris and Wynne in The Canadian problem gambling index: User manual. Canadian Centre on Substance Abuse, Toronto 2001a), the 8-item Brief Sensation Seeking Scale (BSSS-8) (Hoyle et al. Pers Individ Diff 32(3): 401–414, 2002), Bradburn’s Affect Balance Scale (BABS) (Bradburn in The structure of psychological well-being. Aldine, Chicago 1969) and questions on gambling activities. The response rate is 65%. Results indicate 32.3% (*n* = 323) of the survey participants wagered on mahjong (61.8%), soccer matches (40.2%), Mark Six lottery (37.2%), card games (28.1%), land-based casino gambling (13.1%), slot machines (7.5%) and online casino games (2.0%). The average monthly stake was MOP $411. Seeking entertainment (18.7%), killing time (12.5%) and peer influence (11.1%) were the three main reasons for gambling. Using the PGSI, 3.6 and 5.3% of the students could be identified as moderate-risk and problem gamblers respectively. Men were significantly more vulnerable to gambling problems (X2(1) = 35.00, *p* < 0.01) than women. Most of the problematic gamblers (76%) made their first bet before 14 years. The PGSI scores are significantly correlated with the BSSS-8 scores (*r* = 0.23, *p* < 0.01) but not with the overall ABS scores (*r* = −0.06, *p* > 0.05). The study findings inform campus prevention programs and future research.

## Background

Youth is susceptible to gambling involvement and gambling problems. College and university students are a particularly vulnerable group (Koross 2016; Moore et al. 2013; Mubarak and Blanksby 2013; Weinstock et al. 2008). Many students have free time, money, increased freedom, accessibility and interest to play different gambling games. Approximately, 42–80% of college and university students reported gambling in the previous six to twelve months (Burger et al. 2006; LaBrie et al. 2003: Moore et al. 2013; Stinchfield et al. 2006; Weinstock et al. 2007). Oster and Knapp (1998) reported 97% of male college students at the University of Nevada, Las Vegas and 91% of females gambled over the course of their lifetime.

Research data indicate around 6–8 percent of these young people have a serious gambling problem worldwide (Derevensky and Gupta 2007; George et al. 2016; Moore et al. 2013; Mubarak and Blanksby 2013). Nowak (2014) conducted a comprehensive literature review of 72 studies involving 41,989 university students and student athletes worldwide. The estimates of pathological and problem gambling among the university (college) students were 6.13 and 10.23% respectively. Problem gambling would interfere with study, damage relationships and health, cause financial disaster, lead to illegal acts and affect future work prospects.

There is a paucity of research on gambling behavior among the college and university students in Macau where legalized gambling is widely available and accessible. Macau is famed as the Las Vegas of the East. There were 36 casinos in such a small city of only 30.4 square meters in 2015. In the recent decade, the Macau government has provided more resources to support gambling research to increase evidence-based data on Macau citizens’ gambling behavior and problem gambling. For example, prevalence studies on gambling behavior of Macau adolescents and adults were conducted almost every three years to update the estimates of gambling involvement and addiction in Macau. Around 50% of the Macau residents aged 15–64 years gambled in the previous year (University of Macau 2003, 2010, 2013a). The latest government-commissioned study (University of Macau 2013) revealed that 0.9% of the 2158 Macau residents surveyed could be identified as probable pathological gamblers, while 1.9% could be defined as problem gamblers using the DSM-IV criteria.

The past-year estimates of pathological gambling and problem gambling among Macau adolescent high school students are higher than those of the adults, ranging from 5 to 8% (University of Macau 2003, 2010, 2013a; Wong 2009). Our understanding of gambling behavior among the college and university students is inadequate because research is scarce. Current available evidence-based data on Macau university students’ gambling behavior were generated from a very small number of studies.

Wong et al. (2008) conducted a survey targeted 198 Macau undergraduate students (80 males and 118 females) aged 18–24 years. Results showed that 2.5% of the survey participants were problem gamblers. Problem gamblers often experience serious financial, social, emotional and health consequences. Impulsivity was found positively associated with problem gambling, whereas life satisfaction and knowledge about gambling were negatively correlated to problem gambling.

There was another survey aiming to investigate correlates of problem gambling among 952 community adults and 427 university students (Wu et al. 2014a, b). Online gambling was associated with pathological gambling in both the community and student samples. Significant risk factors of pathological gambling were identified, namely the male gender, materialism, casino employment and life dissatisfaction. Wu (2012) also identified predictors of problem gambling in a sample of 932 Chinese college students aged 18–25 years in Macau and Hong Kong. The predictors are gambling intention and perceived control.

Little gambling research among Macau college and university students has been conducted. We hope this study would help filling the research gap to provide empirical data on these students’ gambling behavior. Correlates of problem gambling would also be examined. Previous studies indicated that problem gambling was associated with affect states (e.g. Atkinson et al. 2012; Matthew et al. 2009; University of Macau 2013b) and sensation seeking propensity (Harris et al. 2013; Zuckerman 2007). We would like to examine if affect states and sensation seeking are also risk factors of problem gambling among Macau college and university students. The study results would have implications for campus gambling awareness activities.

Early student studies discovered a link between problem gambling and affect states. Atkinson et al. (2012) conducted a survey among 448 young adult college students. They found that gambling severity was correlated with negative affect. In another student study with 127 Internet gamblers (Matthew et al. 2009), negative mood states after gambling online and negative mood states generally were the best predictors of problem gambling. While agreeing with the linkage between problem gambling and affect states, Hills et al. (2001) argued that the link was not a causal relationship. We wonder if affect states would also play a role in problem gambling among Macau college and university students.

The role of sensation seeking in problem gambling has been verified in several studies but not yet examined in Macau student gambling surveys. Sensation seeking is a personality trait showing an individual’s desire for experiences and feelings which are novel, complex and intense (Zuckerman 2009). The individual may ignore or tolerate potential risk associated with these experiences. Sensation seeking is often linked to thrill seeking activities such as gambling, smoking and drinking. These activities may provide a lot of stimulation and excitement. The high sensation seekers need plenty of stimulation to obtain optimal level of arousal. Problem gambling is commonly found among high sensation seekers (Gupta and Derevensky 1998a; Powell et al. 1999). For example, in a study conducted among youth and adult gamblers, Harris et al. (2013) found that sensation seeking was positively associated with student pathological gambling. Sensation seeking is also identified as a robust predictor of excessive gambling.

To conclude, this study aimed to fill a research gap by providing evidence-based data on gambling behavior and problem gambling among Macau college and university students. We also attempted to identify the correlates of problem gambling because correlation data would inform preventive measures. Previous western studies indicated that sensation seeking and affect states were associated with youth problem gambling. We tried to explore if sensation seeking and affect states would play a role in student gambling problems across culture. To our knowledge, sensation seeking and affect states have never been examined in Macau’s gambling surveys. We expected that student problem gambling would be associated with affect states and sensation seeking propensity. We also expected gambling would be a male dominated activity, and male student gamblers would be more vulnerable to problem gambling than the women student gamblers. The study findings would throw light on campus education and preventive programs.

## Methods

### Procedures

This was a student survey. Ethics approval for conducting the study was granted by the institution’s Training and Research Committee before research launch. In early 2014, letters were sent to all the Macau colleges and universities inviting their students to participate in the study. Explanation about survey aims and procedures was included in the letter. Ten Macau colleges and universities agreed to provide support to the study. With the help provided by the college and university teaching staff, the trained research assistants distributed 1548 survey questionnaires to potential survey participants during the recess. Survey participation was voluntary and students were not asked to provide their names. The students were informed that their responses would be kept confidential. A convenience sample of 999 students aged 17–52 years (mean age = 21.6 years, SD = 3.7) filled and returned the questionnaires with the written consent forms. The response rate is 64.5%.

### Participants

Among the 999 participants, there were 629 women and 370 men aged 17–25 years (mean age = 21.6 years, SD = 3.7). Many were studying in the first year (*n* = 413, 41.3%), 20.2% (*n* = 202) were second year students, 19.0% (*n* = 190) were third year students, and 17.9% (*n* = 179) were fourth year students.

Majority received financial support from their parents (*n* = 707, 70.7%), 45.9% earned an income from jobs (*n* = 459). Only 2.4% (*n* = 24) admitted that some of their income came from gambling activities. Many students (*n* = 399, 39.9%) reported having a monthly income of MOP $1000–$2999, 17.9% (*n* = 179) had more than MOP $10,000 per month, 17.2% (*n* = 172) had MOP $4000–$6999 each month and 15.0% (*n* = 150) had less than MOP $1000 each month.

### Measures

The self-administered questionnaire included the following sections:

1. Demographic information on age, sex, incomes and year of study;
2. Questions on gambling reasons, gambling frequency, monthly amount wagered on gambling, and types of gambling activities preferred in the past year;
3. The 9-item Problem Gambling Severity Index (PGSI) (Ferris and Wynne 2001a) assessed the severity of gambling problems. The PGSI measures gambling involvement, problem gambling and harmful consequences. The PGSI is reliable (Cronbach’s alpha > 0.70) and has good construct validity (Sharp et al. 2012) with a unidimension of problem gambling. The PGSI has been widely used in many countries including the United Kingdom (Orford et al. 2010), Australia (McMillen and Wenzel 2006), Singapore (Arthur et al. 2008) and Canada (Ferris and Wynne 2001b). The students’ responses to the PGSI were rated on a four-point scale (0 = never, 1 = sometimes, 2 = most of the time and 3 = almost always). Total scores ranged from 0 to 27. Higher the PGSI scores, greater the risk of problem gambling. The results were interpreted as follows: (a) a score of “0” implied non-problem gambling, (b) a score of 1–2 indicated low-risk gambling with few or no negative consequences, (c) a score of 3–7 suggested moderate-risk gambling leading to some substantial negative consequences, and (d) a score of 8 or more indicated problem gambling with harmful consequences and a possible loss of control over gambling.
4. Sensation seeking was assessed by the 8-item Brief Sensation Seeking Scale (BSSS-8) (Hoyle et al. 2002). The BSSS-8 is reliable with Cronbach’s alphas ranging from 0.60 to 0.79 (Aluja et al. 2004; Hoyle et al. 2002; Stephenson et al. 2007). The BSSS-8 also has good construct validity. It retains Zuckerman’s (1976, 1994) conceptualization that sensation seeking as a personality trait is composed of four components (Ferrando and Chico 2001), namely thrill and adventure seeking, experience seeking, disinhibition and boredom susceptibility. The scale includes 2 items for each component. Using a four-point scale (1 = strongly disagree, 2 = disagree, 3 = agree, 4 = strongly agree), total scores range from 0 to 32. Higher the BSSS score, greater the desire to seek sensation.
5. The Bradburn’s Affect Balance Scale (ABS) (Bradburn 1969) was used to assess the students’ affect states. There are 10 items in the scale. Five items explore positive affect, while the other five items examine negative affect. The students’ affect states were measured as a net balance of negative affect (5 items) and positive affect (5 items). Participants were asked if they had experienced certain emotions in the previous four weeks (e.g. feeling “bored” or “on top of the world”). Responses were made in a “yes” and “no” format but only each affirmative answer would be given one score. The overall “Affect Balance Score” (ABS) comes from subtracting the negative affect score from the positive affect score. Higher ABS scores suggest more positive affect is being experienced. The ABS has been tested to be a reliable and valid instrument (Bradburn 1969). Internal consistency reliabilities for Negative Affect scores range from 0.61 to 0.73; for Positive Affect scores range between 0.55 and 0.73. Factor analyses indicated that positive and negative affect were distinct dimensions with small associations between them (0.04–0.15).

## Results

### Gambling participation and problem gambling

As shown in Table 1, almost one-third (*n* = 323, 32.3%) of the survey participants (143 men and 180 women) gambled in the past year. Using the PGSI (Ferris and Wynne 2001a), 19.6% (*n* = 196) of the 999 survey participants were identified as non-problem gamblers, 3.8% (*n* = 38) were low-risk gamblers, 3.6% (*n* = 36) were moderate risk gamblers, and 5.3% (*n* = 53) could be classified as problem gamblers.

**Table 1:** Gambling participation and problem gambling among the survey participants (*n* = 999)

Gamblers	Macau students (*n* = 999)	
	N	%
Non-gamblers	676	67.7
Non-problem gamblers	196	19.6
Low-risk gamblers	38	3.8
Moderate-risk gamblers	6	3.6
Problem gamblers	53	5.3

### Gender difference in gambling participation and problem gambling

Since more women than men were recruited in this study, there were more female gamblers (*n* = 180, 55.7%) than male gamblers (*n* = 143, 44.3%) (Table 2). There were also more female low-risk gamblers (*n* = 25, 65.8%) than male low-risk gamblers (*n* = 13, 34.2%). However, more men (*n* = 63) than women (*n* = 26) obtained a score of 3 or more on the PGSI. The threshold of moderate-risk gambling is a score of three. The sex difference in moderate and problem gambling is significant (X2 (1) = 35.0, *p* < 0.01). As shown in Table 2, 61.1% of the moderate-risk gamblers were males, and 38.9% were females. Similarly, 77.4% of the problem gamblers were males, and 22.6% were women.

**Table 2:** Sex difference in gambling participation and problem gambling (*n* = 999)

Gamblers (PGSI score)	Male		Female		Total	
	N	%	N	%	N	%
Non-problem gamblers	67	34.2	129	65.8	196	100
Low-risk gamblers	13	34.2	25	65.8	38	100
Moderate-risk gamblers	22	61.1	14	38.9	36	100
Problem gamblers	41	77.3	12	22.6	53	100
Total	143	44.3	180	55.7	323	100

### Reasons for gambling

The five most frequently reported reasons for gambling were entertainment (*n* = 121, 37.5%), killing time (*n* = 81, 25.1%), peer influence (*n* = 72, 22.3%), affordability due to acceptance of small stakes (*n* = 57, 17.6%) and perceiving gambling activities as a challenge (*n* = 55, 17.0%).

### Preferred gambling forms

The preferred gambling forms were mahjong (38.1%), soccer betting (25.4%), Mark Six lottery (22.9%), card games (17.3%), stocks (9.0%), land-based casino gambling (8.0%), slots (5.0%) and online casino games (1.2%). With the exception of mahjong, men outnumbered women in all these gambling activities.

### Gambling frequency

Among 96 gamblers who answered the question on gambling frequency, 42.7% (*n* = 41) gambled once a month, 20.8% (*n* = 20) played 2–3 times a month, 18.8% (*n* = 18) played 1–2 times a week, 12.5% (*n* = 12) played once a day, 4.2% (*n* = 4) played 3–4 times a week, and one gambler (1.0%) played 5–6 times a week.

### Money wagered on gambling

On average, the gamblers wagered MOP $411 a month on gambling activities. The highest stake reported was MOP $10,000. On average, the gamblers spent 3.88 h (SD = 3.41) on a gambling activity. The longest gambling duration reported by a gambler was 12 h.

### Age of first gambling

Among the 323 gamblers, the mean age of first gambling of was 14.5 years (SD = 4.7). Most of the problematic gamblers (i.e. combining the cases of moderate-risk and problem gamblers) (*n* = 67, 76.1%) placed their first bet before 14 years. Only 23.5% (*n* = 46) of the non-problem gamblers did so. The difference is significant [X2 (2) = 66.1, *p* < 0.01].

### Gambling debts

Only five (1.5%) of the 323 gamblers reported having gambling debts. All of them were problematic gamblers who obtained a score of 3 or more on the PGSI.

### Family problem gambling

Among the fifty-three problem gamblers, twenty (37.7%) reported that their family members had a gambling problem in the previous twelve months, while the majority (*n* = 33, 62.3%) did not have the same problem.

### Sensation seeking among the gamblers

The gamblers’ desire for seeking sensation was measured by the 8-item Brief Sensation Seeking Scale (Hoyle et al. 2002). Higher the BSSS-8 scores, greater the need to seek sensation. As indicated in Table 3, the problem gamblers obtained the highest mean BSSS-8 score of 22.3 (SD = 4.4). The low-risk gamblers’ mean BSSS-8 score is 21.1 (SD = 2.9) which is the second highest score. The non-problem gamblers’ mean BSSS-8 score is 20.1 (SD = 3.4) which is slightly higher than the mean BSSS-8 score of 19.9 (SD = 3.6) obtained by the moderate-risk gamblers. In short, the problem gamblers were most fond of seeking sensation than the other three types of gamblers.

**Table 3:** Student gamblers’ scores on the 8-item Brief Sensation Seeking Scale (*n* = 323)

Gamblers	Mean BSSS scores	SD	t	*p* value
Non-problem gamblers	20.14	3.4	5.1	0.002**
Low-risk gamblers	21.05	2.9		
Moderate-risk gamblers	19.94	3.6		
Problem gamblers	22.3	4.4		

** *p* < 0.01

To enhance further comparison of the mean BSSS-8 scores between the gamblers with and without severe negative consequences, the gamblers were re-classified as the “non-problematic gamblers” (i.e. the cases of non-problem and low-risk gamblers were combined), and the “problematic gamblers” (i.e. the cases of moderate-risk and problem gamblers were combined). The former experienced few or no harmful consequences, and the latter had experienced serious adverse consequences. The mean BSSS-8 score for these two groups of gamblers are 20.3 (SD = 3.3) and 23.1 (SD = 4.2) respectively, implying the problematic gamblers were more fond of sensation seeking than the non-problematic gamblers. The difference is significant (t = 5.1, *p* < 0.01).

### Affect states of the gamblers

Bradburn’s Affect Balance Scale (1969) (ABS) was used to measure the gamblers’ emotional well-being. Higher overall ABS score suggests more positive affect. As shown in Table 4, the low-risk gamblers obtained a mean ABS score of 0.87 (SD = 1.6) which is the highest score found in these four types of gamblers. The non-problem gamblers had a mean score of 0.79 (SD = 1.6) which is the second highest score. The moderate-risk gamblers and the problem gamblers obtained a much lower mean ABS score of 0.50 (SD = 2.0) and 0.67 (SD = 1.7) respectively.

**Table 4:** Student gamblers’ scores on the Bradburn’s Affect Balance Scale (*n* = 323)

Gamblers	Mean ABS scores	SD	*t*	*p* value
Non-problem gamblers	0.79	1.6	0.80	0.06
Low-risk gamblers	0.87	1.6		
Moderate-risk gamblers	0.50	2.0		
Problem gamblers	0.67	1.7		

To enhance further comparison of the mean ABS scores between the gamblers with and without serious negative gambling harms, the gamblers were re-classified as the “non-problematic gamblers” and the “problematic gamblers”. The former experienced no or few adverse gambling harms, while the latter might have experienced serious negative consequences. The mean ABS score for these two groups of gamblers are 0.81 (SD = 1.6) and 0.61 (SD = 1.8) respectively, suggesting that the non-problematic gamblers did experience more positive affect than the problematic gamblers as their mean ABS score is higher but the difference is not statistically significant (t = 0.8, *p* > 0.05).

### Correlates of problem gambling

Table 5 summarizes the correlation findings generated from the Pearson product moment test. Problem gambling was significantly correlated with age of first gambling (*r* = −0.8, *p* < 0.05), age of the gamblers (*r* = 0.24, *p* < 0.01), the BSSS-8 scores (*r* = 0.23, *p* < 0.01) and gambling frequency (*r* = 0.17, *p* < 0.05) but not with the ABS scores (*r* = −0.06, *p* > 0.05).

**Table 5:** Correlations between PGSI scores and other key variables

Variables	PGSI scores
Age of gamblers	0.24**
BSSS scores	0.23**
ABS scores	−0.06
Age of first gambling	−0.8*
Gambling frequency	0.17*

* *p* < 0.05; ** *p* < 0.01

## Discussion

### Gambling participation and problem gambling

The students’ past-year gambling participation rate in this study is only 32.3%. Compared with the rates of 40–50% found in previous Macau studies (e.g. University of Macau 2013a, b; Wu et al. 2014a, b), this gambling participation rate is relatively low. However, the estimate of problem gambling (5.3%) is close to the rates of 6–8% reported in many western studies (Derevensky and Gupta 2007; George et al. 2016; Moore et al. 2013; Mubarak and Blanksby 2013; Nowak 2014).

Interestingly, the rates of problem gambling (5.3%) and moderate-risk gambling (3.6%) found in this survey are slightly higher than those noted in previous Macau studies (Wong et al. 2008; Wu 2012; Wu et al. 2014a, b). For example, Wong et al. (2008) reported that 2.5% of the 198 Macau undergraduates were problem gamblers. Wu and her researchers (2015) also reported a similar problem gambling rate of 2.1% among 427 Macau university students. More research is needed to increase evidence-based data of gambling involvement and problem gambling among Macau college and university students.

### Sex difference and age correlate of problematic gambling

The survey results replicated previous research findings on gender difference in student problematic gambling (i.e. the cases of moderate-risk and problem gambling were combined) (e.g. George et al. 2016; Koross 2016; LaBrie et al. 2003). More male gamblers (*n* = 63) than females (*n* = 26) scored three or more on the PGSI. Men were significantly more vulnerable to problematic gambling than women.

Gamblers’ age is positively associated with the PGSI scores (*r* = 0.23, *p* < 0.01). Older age gamblers had higher risk of problematic gambling because they might have increased freedom from parental supervision to engage in gambling activities. Campus preventive education should target the male and older students.

### Sensation seeking and problem gambling

In line with previous studies (e.g. Gupta and Derevensky 1998a; Harris et al. 2013; Powell et al. 1999), the survey results have confirmed that sensation seeking is positively correlated to problem gambling severity (*r* = 0.23, *p* < 0.01). Problematic gamblers obtained a significantly higher mean BSSS-8 score (mean = 23.1, SD = 4.2) than the non-problematic gamblers (mean = 20.3, SD = 3.3) (t = 5.1, *p* < 0.01). In short, the problematic gamblers exhibited a stronger desire for seeking sensation than the non-problematic gamblers. In Macau it is very convenient for these high sensation seekers to play gambling games at different gambling venues (e.g. the casinos, the pokies halls, the horse racing course) or via the Internet. Gambling is widely available and accessible in Macau. Gambling might also have fulfilled their need for entertainment and killing time.

### Affect states and problem gambling

As expected, the low-risk and non-problem gamblers experienced more positive affect (ABS score = 0.81, SD = 1.6) than the problematic gamblers (ABS score = 0.61, SD = 1.8) but the difference is not statistically significant. However, contrary to past research results (Atkinson et al. 2012; Matthew et al. 2009), problem gambling severity is not correlated to both negative and positive affect states. More research is needed before conclusion can be made as this is the only study to explore the role of affect states in problem gambling among Macau college and university students. Western studies have provided evidence to support a link between problem gambling and negative affect states (Atkinson et al. 2012; Matthew et al. 2009).

### Early gambling and problem gambling

Many previous studies have also documented early gambling as a risk factor of problem gambling (e.g. George et al. 2016; Griffiths 1995; Gupta and Derevensky 1998b; Wong 2010; Wu et al. 2014a, b). The correlation data confirm that early gambling is negatively correlated to problem gambling severity. Among the four significant correlates of problem gambling found in this study, early gambling is the strongest factor of association. Prevention of early and problematic gambling should begin in primary schools. This study discovered that many problematic gamblers (76.1%) wagered with money before 14 years while only 23.5% of the non-problematic gamblers did so. We suggest prevention programs should begin early before adolescence years but should continue after admission to college and university.

### Campus gambling prevention programs

In line with western research data, the problem gambling rate in Macau colleges is around three times greater than that of the general population (e.g. Mubarak and Blanksby 2013; Nowak 2014; Volberg 1998). Problem gambling would disrupt the students’ study and even their future career prospects. However, little attention has been paid to campus gambling problems in Macau. Shaffer et al. (2000) reported that forty percent of the American institutions failed to recognize gambling as a major problem for their students. We find similar situation in Macau. Without evidence-based information on campus gambling problems, it is not a surprise that Macau institution administrators would not believe their students have gambling problems, not to mention developing appropriate measures to address this health hazard on campus. We hope that the high problematic gambling rate found in this study would attract the attention of the government, the institution administrators and the campus student office. They should work together to prevent student gambling problems and to provide professional help to the problematic gamblers.

## Conclusion

The major weakness of this study is the research data were collected by the convenience sampling strategy. Hence, the power to generalize the study results to the population of Macau college and university students would be adversely affected. Future research should consider using random samples if feasible to enhance data representation.

Despite the weakness, the survey has increased our understanding of the students’ gambling involvement and addiction. The college and university students could be identified as a vulnerable group for gambling problems. It seems that Macau Chinese male and older students who have started gambling early to seek sensation are particularly at risk for problematic gambling. The study results confirm that gender, age, early gambling and sensation seeking propensity are common risk factors across culture. Campus prevention programs should target these high risk students whereas primary prevention measures (e.g. information pamphlets, warning messages and awareness seminars) may be beneficial to all the students. There is a paucity of gambling research targeting the college and university students. It is necessary to conduct more research to investigate the college students’ gambling behavior, and to examine their resiliency and risk for gambling addiction. Systematic evaluation of the current preventive programs is also recommended. Only evidence-based information would inform and improve campus education and intervention programs.
